# A new peptide vaccine OCV-501: in vitro pharmacology and phase 1 study in patients with acute myeloid leukemia

**DOI:** 10.1007/s00262-017-1981-3

**Published:** 2017-03-20

**Authors:** Yukio Kobayashi, Toru Sakura, Shuichi Miyawaki, Kazuyuki Toga, Shinji Sogo, Yuji Heike

**Affiliations:** 1 Department of Hematology, National Cancer Center Hospital, 5-1-1 Tsukiji, Chuo-ku, Tokyo, 104-0045 Japan; 2 Leukemia Research Center, Saiseikai Maebashi Hospital, Maebashi, Gunma 371-0821 Japan; 3 Division of Hematology, Tokyo Metropolitan Ohtsuka Hospital, Toshima-ku, Tokyo, 170-8476 Japan; 4 Department of Clinical Research and Development, Headquarters of New Product Evaluation and Development, Otsuka Pharmaceutical Co., Ltd., Minato-ku, Tokyo, 108-8242 Japan; 5 Microbiological Research Institute, Otsuka Pharmaceutical Co., Ltd., 463-10 Kagasuno, Kawauchi-cho, Tokushima, 771-0192 Japan; 6 Immunotherapy and Cell Therapy Service, St. Luke’s International Hospital, 9-1 Akashi-cho, Chuo-ku, Tokyo, 104-8560 Japan; 7 Translational Medicine Department, Phase 1 Group, Exploratory Oncology, Research & Clinical Trial Center, National Cancer Center Hospital, 5-1-1 Teukiji, Chuo-ku, Tokyo, 104-0045 Japan

**Keywords:** OCV-501, Acute myeloid leukemia, WT1, Helper peptide, Immunotherapy, Cancer vaccine

## Abstract

Wilms’ tumor 1 (WT1) is a promising target of new immunotherapies for acute myeloid leukemia (AML) as well as for other cancers. OCV-501 is a helper peptide derived from the WT1 protein. OCV-501 induced OCV-501-specific Type 1 T-helper (Th1) responses dose-dependently and stimulated helper activity of the specific Th1 cells in peripheral blood mononuclear cells from healthy donors. OCV-501 also enhanced the increase in WT1-killer peptide-specific cytotoxic T lymphocytes. OCV-501 stimulated the OCV-501-specific Th1 clones in an HLA class-II restricted manner and formed a complex with HLA class-II protein. OCV-501-specific Th1 clones demonstrated significant OCV-501-specific cytolytic activity against OCV-501-pulsed B-lymphoblastoid cell line cells. Based on the pre-clinical results, phase 1 clinical trial was conducted. The result of this trial suggested that the subcutaneous administration of OCV-501 once weekly for 4 weeks at doses of 0.3, 1, and 3 mg in older patients with AML during complete remission was safe and well tolerated. The maximum tolerated dose was considered to be ≥3 mg. Of the nine subjects enrolled, neither relapse nor blast cells were observed during the study. Immunological responses were observed in OCV-501-specific delayed-type hypersensitivity test. This trial was registered at http://www.clinicaltrials.gov as NCT 01440920.

**Electronic supplementary material:**

The online version of this article (doi:10.1007/s00262-017-1981-3) contains supplementary material, which is available to authorized users.

## Introduction

Acute myeloid leukemia (AML) is the most common leukemia in older adults. Chemotherapy is a standard treatment for patients with AML and is usually divided into remission induction and consolidation therapy. Treatment efficacy and tolerability deteriorate markedly with advancing age. Complete remission (CR) rates in younger patients exceeded 70%, but declined to 50% in older patients [1]. One of the major obstacles to curing AML, particularly in older patients, is its propensity to relapse after the achievement of CR with chemotherapy or hematopoietic stem-cell transplantation [2]. Therefore, new therapeutic strategies for preventing relapse after consolidation therapy for AML are urgently needed. The graft versus leukemia effect associated with allogeneic hematopoietic stem cell transplantation strongly suggests that immunotherapy is a promising AML treatment [3, 4]. Recent studies have identified several promising AML antigens as targets of immunotherapy [4]. Wilms’ tumor 1 (WT1) antigen is acknowledged as a top-ranked among 75 cancer antigens [5].

The *WT1* was first isolated from Wilms’ tumor, a cancer of the kidney in children, as a tumor suppressor gene [6]. Extensive investigations by Oka and Sugiyama revealed that WT1 possesses oncogenic function and is strongly expressed in hematological malignancies and some solid cancers [7–9]. High-level expression of *WT1* was an accurate predictor of poor disease-free and overall survival rates [10]. The *WT1* antisense oligomers [11] and WT1-specific CTLs [12] inhibited the growth of leukemic cells without affecting normal cells, suggesting that WT1 plays an important role in leukemogenesis.

Clinical trials of cancer vaccines using synthetic WT1 peptide have been conducted in patients with AML as well as with solid tumors for more than a decade and some clinical responses and benefits have been observed [13–15]. The earlier generation of WT1 peptide vaccines was the HLA class I-binding short peptide (killer peptide), consisting of 8–9 amino acids, which can be easily synthesized as a drug candidate. CD8^+^ T cells recognize tumor-associated antigen (TAA)-derived killer peptides presented on cancer cell surfaces in association with HLA class I molecules, leading to cancer cell death [16, 17]. Recently, it was reported [18, 19] that the beneficial effects derived from WT1-killer peptides were short because of the induction of T cell tolerance. Repeated delivery of killer peptides led to the rapid loss of high-avidity peptide-specific CD8^+^ CTLs and CD4^+^ Type 1 T-helper (Th1) cells are required for secondary expansion and memory in CD8^+^ CTLs [20, 21]. Therefore, to overcome poor clinical outcomes of cancer vaccination, helper peptides that elicit CD4^+^ Th1 cells should be considered [22, 23].

OCV-501 (developed by Otsuka Pharmaceutical Co., Ltd., Tokyo, Japan) is a synthetic peptide consisting of the natural sequence derived from the WT1 gene product protein, without any modification or combination with other peptide sequences [24]. It can induce specific CD4^+^ Th1 cells from peripheral blood mononuclear cells (PBMC) from healthy donors, but does not directly induce CD8^+^ CTL with killer epitope-specificity (e.g. WT1-126, WT1-235, etc.). Therefore, OCV-501 is an HLA class II- restricted WT1-helper peptide for therapeutic cancer vaccine. Here, we report in vitro pharmacological properties of OCV-501 and the phase 1 clinical trial in older patients with AML.

## Materials/patients and methods

OCV-501 used in this study was synthesized in Otsuka Pharmaceutical Co., Ltd., Tokyo, Japan (KRYFKLSHLQMHSRKH, purity >95% as acetic acid salt).

### Pre-clinical pharmacology

#### Induction and activation of OCV-501-specific Th1 cells from peripheral blood mononuclear cells

Induction of OCV-501-specific T lymphocytes was performed according to a previously reported method [24]. Briefly, after written informed consent, human PBMC were isolated from 20 healthy donors with one or more of the HLA class II types (DRB1*04:05/15:01/15:02 and DPB1*09:01/05:01) by gradient centrifugation on Lymphoprep (Axis Shield Diagnostics Ltd., Dundee, Scotland). PBMC were cultured with medium mixture of 45% RPMI-1640 (Sigma–Aldrich, St. Louis, USA) + 45% AIM-V (Thermo Fisher Scientific, Waltham, USA) + 10% human AB serum (MP Biomedicals Inc., Santa Ana, US) containing 20 μg/mL of OCV-501 (purity ≥98%) and 10 ng/mL of IL-7 (PeproTech, Inc., Rocky Hill, USA) at 37 °C, 5% CO_2_ (day 0), and the control group was cultured without OCV-501. PBMC were restimulated and cultured with OCV-501-pulsed antigen-presenting cell (APC), which were prepared from PBMC pre-cultured with 20 μg/mL of OCV-501 followed by 50 μg/mL of mytomycin (Kyowa Hakko Kirin Co., Ltd., Tokyo, Japan) in the presence of IL-7 (day 7). From day 9, IL-2 (PeproTech, Inc., Rocky Hill, USA) was added to the culture (final concentration 20 U/mL) at 2-day intervals. The resulting OCV-501-specific Th1 cells were counted by intracellular IFN-γ staining on days 0, 7, and 14. At each time point, the harvested cells were re-stimulated with/without OCV-501 for 6 h, followed by 2 h incubation with Brefeldin A (BioLegend Inc., San Diego, USA), because the cultured T cells were HLA class II^+^. The cells were stained with PE-anti-human CD4 Ab and FITC-anti-human CD8 Ab (Beckman Coulter Inc., Brea, USA). Intracellular IFN-γ staining was then performed according to the manufacturer’s protocol using the BD Cytofix/Cytoperm Fixation/Permeabilization Kit (BD Biosciences, Franklin Lakes, USA. Finally, stained cells were analyzed using Coulter EPICS XL-MCL Flow Cytometer (Beckman Coulter Inc., Brea, USA). Proportions of CD4^+^/intracellular IFN-γ^+^ cells (%) [A] and OCV-501-specific Th1 cells (%) [B] were calculated as follows: [A] = [number of CD4^+^/intracellular IFN-γ^+^ cells]/[total CD4^+^ cell number] × 100, and [B] = A [OCV-501 restimulated] − A [Background: Solvent restimulated], respectively. For HLA class II-blocking assay, the OCV-501-specific T cells induced by a 14-day culture of PBMC with OCV-501 were pre-treated with 10 μg/mL of each antibody; anti-HLA-DR Ab (BD Biosciences, Franklin Lake, USA), anti-HLA-DQ Ab (Beckman Coulter Inc., Brea, USA), or control Ab (mouse IgG2a) (BioLegend, San Diego, USA) for 30 min, and cultured with/without OCV-501 for 24h. Then, produced IFN-γ was measured using BD OptEIA ELISA Sets (human IFN-γ) (BD Biosciences, Franklin Lakes, USA) and VMAX Microplate Reader (Molecular Devices, Sunnyvale, USA). The dose-dependent activation with OCV-501 was evaluated by restimulation of the cultured cells (OCV-501-induced, day 14) with a dose series of OCV-501 (0.1, 1, 10, 100, and 1000 μg/mL).

#### CD8^+^ killer T cell activation by OCV-501-specific Th1 cell

OCV-501-specific Th1 cells and WT1-killer peptide-specific CTLs were induced by 14 days of co-culture of human PBMC from healthy donors with both HLA class I (A*02:01 or A*24:02) and one or more of the HLA class II types (DRB1*04:05/15:01/15:02 and DPB1*09:01/05:01) with medium containing 20 μg/mL of OCV-501 and either 20 μg/mL of WT1-killer peptide WT1-126 [16] (RMFPNAPYL, purity ≥98%, Otsuka Pharmaceutical Co., Ltd.) or WT1-235mu [25] (CYTWNQMNL, purity ≧94%, Otsuka Pharmaceutical Co., Ltd.). The cultured T cells were then plated and cultured with/without OCV-501 in the presence of WT1-killer peptide-pulsed APC (day 0). The number of WT1-killer peptide-specific CTLs was measured by tetramer assay. Briefly, harvested cells (day 0 or day 5) and PE-conjugated tetramer; WT1A*0201 tetramer (for detection of WT1-126-specific CTL), WT1 (mutant) A*2402 tetramer (for detection of WT1-235mu-specific CTL), A*0201 negative tetramer or A*2402 negative tetramer (Medical & Biological Laboratories Co., Ltd., Nagoya, Japan), were mixed vigorously and incubated for 30 min. FITC-conjugated anti-human CD45 Ab (Beckman Coulter Inc., Brea, US) and PC5-conjugated anti-human CD8 Ab (0.5 μL) were added, mixed vigorously and incubated for 15 min. Cells were re-suspended and counted by Coulter EPICS-XL MCL Flow Cytometer.

#### HLA class II-restriction and cytolytic activity of OCV-501-specific Th1 clones

OCV-501-specific Th1 clones (CloneR45-1 and CloneP51-5) were established using the induction/cloning culture with OCV-501 from the PBMC of 2 healthy donors as described above. Each Th1 clone bearing HLA-DRB1*04:05 or HLA-DPB1*05:01 was stimulated by various B-lymphoblastoid cell lines (B-LCL) (RIKEN Cell Bank, Tsukuba, Japan) as APC, which were pulsed with/without OCV-501. Features of HLA class II of the B-LCL used were DRB1*04:05(+), DRB1*04:05(−), DPB1*05:01(+), and DPB1*05:01(−). After APC stimulation, the concentrations of IFN-γ were measured by ELISA.

Complex formation of OCV-501 with HLA class II proteins such as DRB1*01:01, DRB1*04:05, DRB1*08:03, DRB1*09:01, DRB1*15:01, DRB1*15:02, DRB4*01:01 was examined by HPLC method according to the method reported by Sato [26]. Briefly, folding reaction buffer solution was prepared by combining the HLA proteins with OCV-501, positive control peptide; HLA-DRB1*01:01 human CLIP103-117 peptide (PVSKMRMATPLLMQA) or negative control peptide; irrelevant peptide (NELSGEAHKDALGKLY) (MBL, Nagoya, Japan), and folding buffer. The folding reaction buffer solution was incubated at 37 °C overnight, and then analyzed using HPLC with Superdex 200 column (GE Healthcare, Tokyo, Japan) to determine retention time of the HLA proteins.

The cytolytic activities of OCV-501-specific Th1 clones were estimated using the ^51^Cr-release assay [12]. The OCV-501-specific Th1 clones (6 clones) were established from 5 healthy donors (Clone R152-2 and P91-1 were from the same donor), and each clone was confirmed to have one HLA-class II restriction using various HLA-class II-bearing peptide-pulsed/un-pulsed B-LCLs. OCV-501-specific Th1 clones (effector) and ^51^Cr-labeled B-LCLs pulsed with/without OCV-501 (target) were tested at various effector/target (E/T) ratios (40:1, 20:1, and 10:1). At the end of the culture period, 50 μL of each supernatant was collected and radioactivity was calculated using TopCount NXT™ (PerkinElmer, Waltham, US). The percentage of specific lysis of target cells was determined as follows: (experimental count − spontaneous count)/(total count − spontaneous count) × 100 (%).

### Clinical study

#### Study design

This was an open label, multi-center, phase 1 trial. A traditional 3 + 3 study design was used, with cohorts of three to six patients. If one of the three patients experienced a dose-limiting toxicity (DLT) in the cohort, up to three patients would be enrolled at the same dosage level. If two or more patients experienced a DLT, no further dose escalation would be performed and additional patients were to be enrolled at a lower dose, to confirm the maximum tolerated dose (MTD). The MTD was defined as the highest dose at which none of the first three patients or one of up to six patients of total experienced a DLT in the cohort.

#### Patients

Older patients (≥60 years) with AML participated in this study. The eligibility criteria were that patients must have achieved their first CR with an induction regimen and completed standard consolidation therapy, and have been identified as *WT1* mRNA positive, with one of the following HLA class II types: HLA-DRB1*01:01, *04:05, *15:01, *15:02, *08:03, or *09:01. Patients with myelodysplastic syndrome apparently evolved into AML and patients with AML accompanied by t(15;17)(q22;q12), (PML/RARalpha) were excluded. Patients scheduled for bone marrow transplantation, taking immunosuppressants and adrenal cortical steroids exceeding the acceptable therapeutic doses, with autoimmune diseases or with a medical history of active autoimmune diseases, and immunocompetent patients were excluded. HLA genotyping was performed using a PCR-based typing method [27].

#### Drug administration

OCV-501 emulsified with Montanide ISA 51 adjuvant for injection (Seppic Inc., Paris, France) was administered subcutaneously weekly for 4 weeks (day 1, day 8, day 15, and day 22). In each cohort, the first administration of the subsequent patient was allowed only after the second administration of the first patient had been completed. End of treatment and post-treatment examinations were performed after 1 week (on day 29) and 4 weeks (on day 50) from the last administration of OCV-501, respectively. The trial consisted of 3 cohorts at a dose of 0.3 mg in cohort 1, 1 mg in cohort 2, and 3 mg in cohort 3 which is in the range of 0.1 to 10 mg/body that is generally known to have no sign of dose dependency in immunological evaluations [28]. Administration commenced with cohort 1 and progressed to cohorts 2 and 3, depending on the assessment for DLT in the preceding cohort.

#### Safety and efficacy assessments in patients

Safety and tolerability of OCV-501 were operationalized as adverse events, clinical laboratory test, Eastern Cooperative Oncology Group performance status, vital signs (blood pressure, pulse rate, body temperature), body weight, 12-lead ECG, pulse oximetry, chest X-ray, and observation of the administration site. These adverse events were defined as treatment-emergent adverse events (TEAEs) and determined using the National Cancer Institute Common Terminology Criteria for Adverse Events score, version 4.0. DLT was the primary endpoint and determined based on adverse events.

As the efficacy variables, outcomes were evaluated by relapse of AML when assessed according to the International Working Group response criteria [29]. To monitor the minimal residual disease, *WT1* mRNA level in peripheral blood was measured on day 1 (screening) and day 29 (end of treatment) and then once a month until morphological relapse. Peripheral blood levels of *WT1* mRNA were measured using a *WT1* mRNA Assay Kit ‘Otsuka’ (Otsuka Pharmaceutical Co., Ltd., Tokyo, Japan). RNA was extracted using the QIAamp RNA Blood Mini Kit (QIAGEN GmbH, Hilden, Germany) and the PCR product level of *WT1* was quantified. The cut-off value of *WT1* mRNA for early detection of relapse of AML was 200-copy/μg RNA. If the value surpassed the cut-off value, the investigator judged the need for bone marrow examination based on the subject’s condition.

WT1 mRNA level, delayed-type hypersensitivity (DTH), anti-OCV-501 Ab titer, and total IgG were determined as exploratory endpoints. The immune response to OCV-501 was analyzed using a DTH skin reaction test at the screening visit (baseline), on day 29, and day 31. An aqueous solution of OCV-501 (0.1 mg) for injection without Montanide was administered intra-dermally at approximately the center of the forearm flexors of the subject, and redness and induration were assessed at 48 h after administration. Anti-OCV-501 Ab titer was evaluated experimentally using anti-OCV-501 IgG (Otsuka Pharmaceutical Co., Ltd., Tokyo, Japan) in plasma by ELISA on day 1 and day 29. Total IgG was measured on day 1, day 29 and day 50 (post treatment) by immunoassay.

### Statistical analysis

An ANOVA with repeated measures (crossover type) or Wilcoxon signed rank test was performed for proportion assessment between the groups. Dose dependencies were analyzed using the Dunnett test with randomized block design. A two-way ANOVA was performed on specific lysis data with the group and E/T ratio as factors. A two-tailed *t* test was used and the level of significance was set at 0.05. SAS software (release 9.1, SAS Institute Japan) was used for all analyses. Statistical analyses in clinical study were not performed.

## Results

### Pre-clinical pharmacology

#### Ex vivo induction and activation of OCV-501-specific helper T cells

**Fig. 1:**
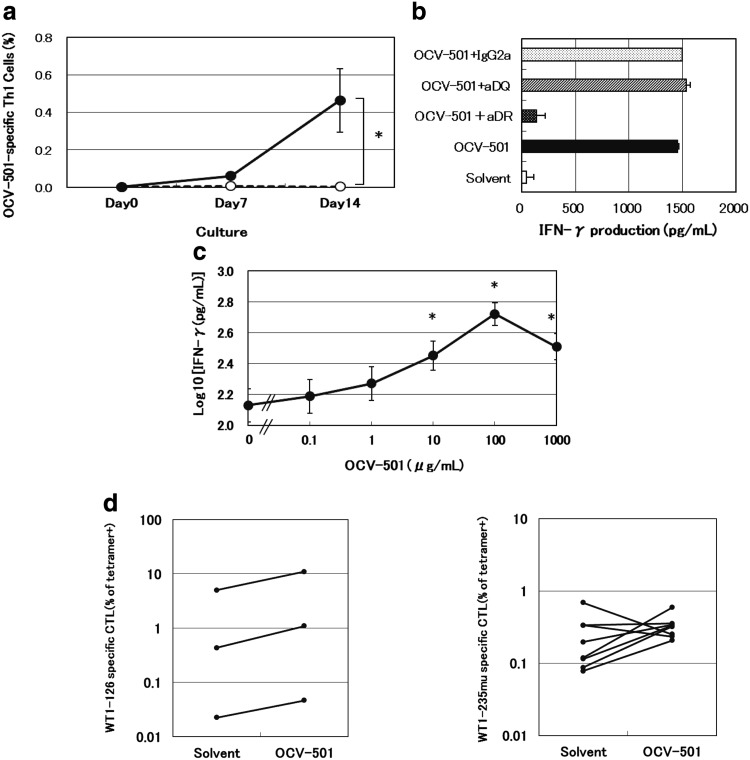
Efficacy pharmacology studies of OCV-501 using PBMC from healthy donor. Th1: Type 1 T-helper, WT1: Wilms’ tumor 1. Induction of OCV-501-specific Th1 cells in PBMC (**a**). PBMC were cultured with OCV-501 (*filled circle*) or solvent (*circle*) (data are expressed as mean ± SE: *n* = 20, *P = 0.0058: main effect in mixed model for repeated measures method, crossover type). The HLA class II-restriction of OCV-501-derived antigen stimulation was evaluated using OCV-501-specific T cells induced in PBMC by blockade with anti-HLA class II antibody (**b**) (data are expressed as mean ± SD, triplicates). Dose-dependence of OCV-501 activation of specific T cells (**c**) (data are expressed as mean ± SE: *n* = 20, *P < 0.05: randomized block design, Dunnett’s test). WT1-specific CTLs were increased by OCV-501 and OCV-501-specific T cells: changes in the number of *WT1*-126-specific CTLs following addition of OCV-501 (**d**) (data are expressed as proportions of 3 independent samples). Changes in the number of WT1-235mu-specific CTLs following addition of OCV-501 (**e**) (data are expressed as proportions of 8 independent samples)

From day 7 to day 14, OCV-501-specific Th1 cells were significantly expanded in OCV-501 culture using human PBMC of 20 healthy donors as shown in Fig. 1a, but not in solvent culture (*p* = 0.0005). Among 18 of 20 PBMC cultures, OCV-501-specific Th1 cells were increased and showed HLA-class II restriction by blocking experiments with anti-HLA-DR Ab. However, these were blocked with neither anti-HLA-DQ Ab nor mouse IgG2a. The HLA-DR restriction was detected in 7 of 18 samples. HLA-DR-specific inhibition in a typical and representative data is shown in Fig. 1b. T-cell activation in the other 11 of 18 samples was not inhibited by anti-HLA-DR/anti-HAL-DQ antibodies. This suggests that OCV-501 would stimulate some types of its specific T-cells in HLA-DR-restricted, and others in HLA-DP-restricted manner. Significant dose-dependent T-cell activation was observed at 10 μg/mL and higher concentrations of OCV-501 in the 20 OCV-501-induced PBMC cultures (Fig. 1c). Helper activity of OCV-501 was confirmed using WT1-killer peptides. OCV-501 enhanced the increase in WT1-126-specific CTLs in 3 out of 3 samples (Fig. 1d) and WT1-235mu-specific CTLs in 6 of 8 samples (Fig. 1e) in the presence of PBMC-derived OCV-501-specific Th1 cells and WT1-killer peptide (WT1-126 or WT1-235mu)-specific CTLs.

#### HLA class II-restriction and cytolytic activity of OCV-501-specific Th1 clones

**Fig. 2:**
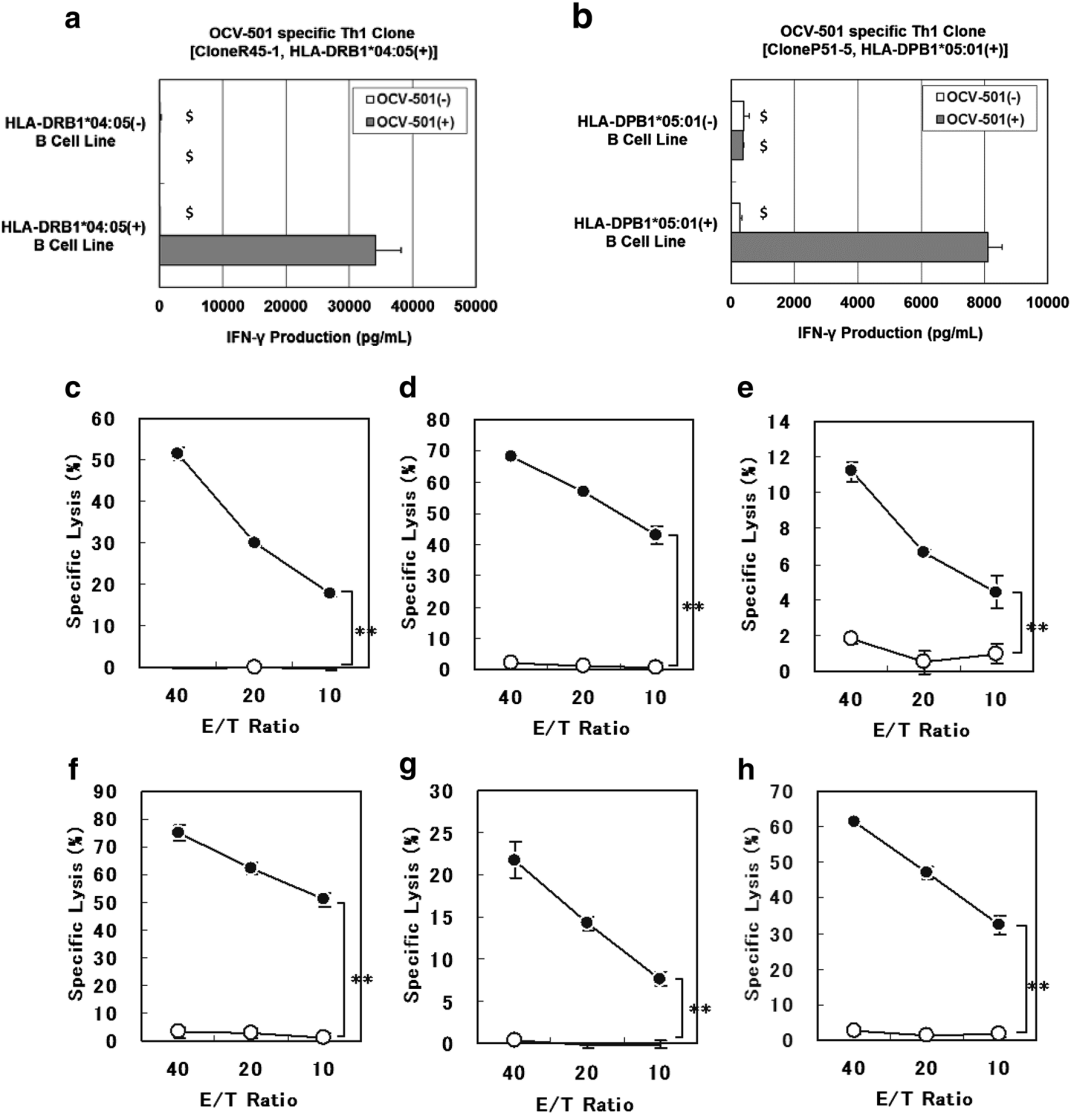
Efficacy pharmacology studies of OCV-501 using various T cell clones. *B-LCL* B-lymphoblastoid cell line, *Th1* Type 1 T-helper. HLA-restricted T-cell activation by OCV-501 using various human T-cell clones. As an index of T-cell activating effect, the produced IFN-γ from OCV-501-specific Th1 clone was estimated in the culture of HLA-DRB1*04:05(+) or DRB1*04:05(−) B-LCL cells pulsed with or without OCV-501 [CloneR45-1: HLA-DRB1*04:05(+) (**a**), CloneP51-5: HLA-DPB1*05:01(+) (**b**)] (data are expressed as mean ± SD, triplicates, $: error bar was calculated by extrapolated data). Cytolytic activity of OCV-501-specific Th1 clones with various HLA class II-restriction (DRB1*04:05 restricted CloneR45-1: (**c**), DRB1*15:02 restricted CloneR152-2: (**d**), DRB1*15:02 restricted CloneR152-3: (**e**), DRB3*02:02 restricted CloneR322-4: (**f**), DPB1*05:01 restricted CloneP51-2: (**g**), DPB1*09:01 restricted CloneP91-1: (**h**)). For specific lysis, each OCV-501-specific Th1 clone was used as effector cells and B-LCL cells pulsed (*filled circle*) or not pulsed (*circle*) with OCV-501 were used as target cells (data are expressed as mean ± SD of triplicates, **P < 0.01, 2-way ANOVA, group effect: *n* = 3)

OCV-501-specific Th1 clone, CloneR45-1, which was bearing HLA-DRB1*04:05 (+), produced IFN-γ, when stimulated with HLA-DRB1*04:05 (+) B-LCL as APC, which were pulsed with OCV-501, but not when stimulated with HLA-DRB1*04:05 (−) B-LCL (Fig. 2a). Similarly, IFN-γ production was only observed in OCV-501-specific Th1 clone, CloneP51-5, which were bearing HLA-DPB1*05:01 (+), when stimulated with HLA-DPB1*05:01 (+) B-LCLs as APC which were pulsed with OCV-501 as shown in Fig. 2b. Moreover, OCV-501 stimulated the OCV-501-specific Th1 clones in an HLA class II-restricted manner with various B-LCLs as APCs bearing the different HLA class II types, such as HLA-DRB1*01:01, DRB1*08:02, DRB1*08:03, DRB1*13:02, DRB1*14:03, DRB1*14:05, DRB1*15:02, DRB3*02:02, DQB1*04:01, and DPB1*09:01 (Supplementary Fig. 1). The HPLC retention time of each HLA protein was markedly delayed more than 0.1 min by mixing with OCV-501 as well as a positive control, while the addition of negative peptide did not change the retention time (Table 1). This suggested complex formation of OCV-501 with the protein products of HLA class II DRB1*09:01, DRB1*15:01, DRB1*15:02, and DRB4*01:01 alleles. The cytolytic activities of OCV-501-specific Th1 clones (6 clones) were estimated using the ^51^Cr-release assay. Significant OCV-501-specific cytolytic activities against OCV-501-pulsed and restriction-matched B-LCL cells were found in OCV-501-specific Th1 clones (Fig. 2c–h).

**Table 1:** Complex formation of HLA class II/peptide

HLA class II	Formation of complexes with various HLA class II molecules (change in HPLC retention time : Δmin)^a^					
	Negative control^b^		Positive control^c^		OCV-501	
DRB1*04:05	−	(0.021)	+	(0.461)	+	(0.470)
DRB1*15:01	−	(0.076)	+	(0.410)	+	(0.278)
DRB1*15:02	−	(0.001)	+	(0.298)	+	(0.371)
DRB1*01:01	−	(0.028)	+	(0.418)	+	(0.525)
DRB1*08:03	−	(0.007)	+	(0.412)	+	(0.538)
DRB1*09:01	−	(0.007)	+	(0.285)	+	(0.325)
DRB4*01:01	−	(0.034)	+	(0.132)	+	(0.180)

^a^Negative control (16-mer:NELSGEAHKDALGKLY)

^b^Positive control (15-mer-CLIP:PVSKMRMATPLLMQA)

^c^(<0.1), + (≥0.1)

#### Clinical trial

Between October 2011 and February 2013, 13 patients from 4 study sites in Japan were enrolled in this trial. Of the 13 patients from whom written informed consent was obtained, 4 were specified as screen failures (2 patients did not match the HLA types in inclusion criteria, 1 patient had Grade 3 lab test abnormality and 1 patient had a relapse of AML). Nine evaluable patients were enrolled in this study (Table 2). The median age was 70 years (range, 62─74 years). Chemotherapy varied among these patients, however, all had completed the planned AML therapy at the time of vaccination and were in first CR according to standard criteria. All had evidence of a measurable *WT1* transcript at screening. Each cohort consisted of 3 subjects and all subjects completed the planned 4 vaccinations with either a 0.3, 1 or 3 mg dose of OCV-501.

**Table 2:** Demographics

Dose	Subject ID	Classification of acute myeloid leukemia	Sex	Age (years)	HLA-DRB1 Genotype		ECOG PS
					Result 1	Result 2	Score^a^
0.3 mg	001–0001	Acute myeloid leukemia, not otherwise specified, AML without maturation	Female	70	08:03	09:01	0
	002 − 0001	Acute myeloid leukemia, not otherwise specified, AML with maturation	Female	62	04:05	04:06	0
	002–0002	Acute myeloid leukemia, not otherwise specified, AML with minimal differentiation	Male	69	13:02	15:01	0
1.0 mg	001–0002	Acute myeloid leukemia, not otherwise specified, AML without maturation	Female	65	01:01	15:02	0
	002–0003	Acute myeloid leukemia with recurrent genetic abnormalities, AML with t(8;21)(q22;q22); RUNX1-RUNX1T1	Male	71	04:05	09:01	0
	002–0004	Acute myeloid leukemia with recurrent genetic abnormalities, AML with inv(16)(p13.1q22) or t(16;16)(p13.1;q22); CBFB-MYH11	Female	72	09:01	14:54	0
3.0 mg	001–0003	Acute myeloid leukemia, not otherwise specified, AML with maturation	Male	74	09:01	13:02	1
	001–0004	Acute myeloid leukemia, not otherwise specified, AML with maturation	Male	73	04:05	04:06:01	0
	002–0005	Acute myeloid leukemia, not otherwise specified, AML with maturation	Male	62	04:07	15:02	0

*AML* acute myeloid leukemia, *HLA* human leukocyte antigen, *ECOG PS* Eastern Cooperative Oncology Group, performance status

^a^0: Fully active, able to carry on all pre-disease performance without restriction

1: Restricted in physically strenuous activity, but ambulatory and able to carry out work of a light or sedentary nature, e.g., light house work, office work

2: Ambulatory and capable of all self-care, but unable to carry out any work activities. Up and about more than 50% of waking h

3: Capable of only limited self-care, confined to bed or chair more than 50% of waking h

4: Completely disabled. Cannot carry on any self care. Totally confined to bed or chair

#### Safety assessment

Incidence of TEAEs is summarized in Table 3. There were neither deaths nor serious TEAEs during the treatment period. None of the subjects discontinued OCV-501 administration due to TEAE. Grade 3 TEAEs included lymphocyte count decreased and neutrophil count decreased (1 subject each) in the OCV-501 1-mg cohort and thrombocytopenia (1 subject) in the OCV-501 3-mg cohort. These events were considered unrelated to OCV-501. The incidence of drug-related TEAEs by system organ class and preferred term are presented in Table 3. Injection site reactions, including erythema, induration, mass, pain, and pruritus were observed in all subjects. However, all drug-related TEAEs were Grade 1 or 2 in severity. No DLT was observed in any of the 3 cohorts and the MTD was considered to be ≥3 mg. This suggests that OCV-501 is safe and tolerable.

**Table 3:** Incidence of drug-related treatment-emergent adverse events

System organ class Preferred term	0.3 mg			1 mg			3 mg			Total	
	Grade 1	Grade 2	Grade 3–5	Grade 1	Grade 2	Grade 3–5	Grade 1	Grade 2	Grade 3–5		
	*N* = 3	*N* = 0	*N* = 0	*N* = 3	*N* = 0	*N* = 0	*N* = 3	*N* = 1	*N* = 0	*N* = 9	
	*N*	*N*	*N*	*N*	*N*	*N*	*N*	*N*	*N*	*N*	(%)
General disorders and administration site conditions	3	0	0	3	0	0	3	0	0	9	(100.0)
Injection site erythema	3	0	0	3	0	0	3	0	0	9	(100.0)
Injection site induration	2	0	0	2	0	0	3	0	0	7	(77.8)
Injection site mass	1	0	0	0	0	0	0	0	0	1	(11.1)
Injection site pain	0	0	0	1	0	0	0	0	0	1	(11.1)
Injection site pruritus	1	0	0	2	0	0	0	0	0	3	(33.3)
Investigations	0	0	0	1	0	0	0	1	0	2	(22.2)
Eosinophil count increased	0	0	0	1	0	0	0	0	0	1	(11.1)
Lymphocyte count decreased	0	0	0	0	0	0	0	1	0	1	(11.1)
Neutrophil count decreased	0	0	0	0	0	0	0	1	0	1	(11.1)
White blood cell count decreased	0	0	0	0	0	0	0	1	0	1	(11.1)
Musculoskeletal and connective tissue disorders	1	0	0	0	0	0	0	0	0	1	(11.1)
Joint swelling	1	0	0	0	0	0	0	0	0	1	(11.1)
Nervous system disorders	1	0	0	1	0	0	0	0	0	2	(22.2)
Headache	0	0	0	1	0	0	0	0	0	1	(11.1)
Hypoaesthesia	1	0	0	0	0	0	0	0	0	1	(11.1)
Skin and subcutaneous tissue disorders	1	0	0	0	0	0	0	0	0	1	(11.1)
Erythema	1	0	0	0	0	0	0	0	0	1	(11.1)

MedDRA version 14.0

#### Efficacy outcomes

None of the 9 subjects with AML had a relapse from the time of screening (day-14 to day-1) to the time of end-of-trial (day 29). In the blood smear examination, no blast cells were observed in any of the 9 subjects. The percentage of myeloblasts in the 9 subjects was less than 5% before and after OCV-501 vaccination (Supplementary Table 1). Expressed levels of *WT1* mRNA at screening (day 1) and at the end of treatment (day 29) were <50–950 and <50–2400 copies/µg RNA, respectively (Table 4). Decreases in *WT1* mRNA were found in 2 subjects in the OCV-501 1-mg cohort and 2 subjects in the 3-mg cohort. All subjects were tested for a DTH response. Redness with induration of >5 mm diameter was confirmed in 1 subject each in the 0.3-mg cohort and 3-mg cohort and redness without induration was observed in 2 subjects in the 0.3 mg cohort and 1 subject each in the 1-mg cohort and 3-mg cohort (Supplementary Table 2). Anti-OCV-501 IgG was negative in all subjects at end of treatment (day 29) (Table 4). Total IgG levels at screening (day 1), end of treatment (day 29), and post treatment (day 50) were 1097–1499, 1041–1601, and 1106–1725 mg/dL, respectively (Supplementary Table 3).

**Table 4:** Immunologic response

Dose	Subject ID	Time point	*WT1* mRNA (copy/µg RNA)	Anti-OCV-501 antibody concentration (ng/mL)
0.3 mg	001–0001	Day 1	<50	Negative
		End of treatment	75	Negative
	002 − 0001	Day 1	950	Negative
		End of treatment	2400	Negative
	002–0002	Day 1	<50	Negative
		End of treatment	66	Negative
1.0 mg	001–0002	Day 1	<50	Negative
		End of treatment	57	Negative
	002–0003	Day 1	64	Negative
		End of treatment	<50	Negative
	002–0004	Day 1	120	Negative
		End of treatment	69	Negative
3.0 mg	001–0003	Day 1	150	Negative
		End of treatment	120	Negative
	001–0004	Day 1	340	Negative
		End of treatment	<50	Negative
	002–0005	Day 1	51	Negative
		End of treatment	59	Negative

*WT1* Wilms’ Tumor 1 gene

## Discussion

OCV-501 is a synthetic helper peptide, which consists of the natural sequence derived from the WT1 protein [24]. It was demonstrated that OCV-501 significantly induced OCV-501-specific Th1 cells in PBMC from 20 healthy donors. Using both the induced primary Th1 cells and the established Th1 clones, OCV-501 also activated the specific Th1 cells in a dose-dependent manner. Furthermore, OCV-501 increased WT1-killer peptide-specific CTLs in the presence of OCV-501-specific Th1 cells. OCV-501-specific Th1 clones demonstrated significant OCV-501-specific cytolytic activity against OCV-501-pulsed B-LCL cells. In addition, reports have shown that WT1_332_ (a WT1 helper peptide that has the same amino acid sequence as OCV-501)-specific Th1 clone and -specific TCR-transduced CD4^+^ T cells were able to respond to WT1-transfected B-LCL and kill WT1^+^ leukemic cells, respectively [24, 30, 31]. It has been reported that HLA-DR molecules are highly expressed in AML cells [32]. Therefore, these pre-clinical results suggest that OCV-501 activates both direct and indirect antitumor (anti-leukemic) cellular immunity, including specific cytotoxic Th1 cells and WT1-peptide-specific CTL cells, however, it is necessary to confirm the cytolytic activity of OCV-501-specific Th1 cells against several AML-derived leukemic cell lines.

HLA class II-restriction of helper peptides is generally not as strict as the HLA class I-restriction of killer peptides, since helper peptides can bind to a variety of HLA class II molecules with loose recognition [33]. In this study, OCV-501 also showed binding to and/or activation of T cell via at least 15 HLA class II molecules. Expression frequencies of these HLA class II molecules in Asian including Japanese, Caucasian, and African black were 83.8–98.8, 79.2–80.2, and 67.7–68.8%, respectively [34]. This suggests that OCV-501 can be used not only for Japanese patients but also for worldwide cancer patients.

Much attention has been paid to the development of helper peptides [22, 23], since helper peptide vaccination induces and activates cancer-specific Th1 cells, which are the control tower for cancer immunity, and increases the antitumor effect by inducing the antibodies that stimulate proliferation and activation of CTLs, activation of APCs, and incorporation of cancer cells through IL-2 and IFN-γ production. Several approaches have been investigated to potentiate the clinical responses of killer peptide-based vaccines [35, 36]. Mixture of killer and helper peptides and a long helper peptide containing class I and class II epitope regions demonstrated to respond to both CD4^+^ and CD8^+^ T cells [17, 37–42]. Potential clinical benefits of the multiple peptide vaccine were also observed in survival curves in patients with AML compared with unvaccinated controls [40]. These observations suggest that the multiple peptides including killer/helper epitopes seems to be one of the promising strategies for activating both CD4^+^ Th1 and CD8^+^ CTL responses. On the other hand, vaccination with a mixture of 6 HLA-DR- restricted melanoma helper peptides induced both specific Th1-dominant CD4^+^ T cell responses and Ab responses, associated with improved overall survival among patients with metastatic melanoma [43, 44]. The survival strongly correlated with early Ab response and/or with early T-cell response [44]. There was a hierarchy of immunodominance of helper peptides and no evidence that peptide length or the type of source tumor antigen predicted immunodominance. These results would reveal that only one single immunodominant helper peptide can elicit both specific Th1-dominant CD4^+^ T cell responses and Ab responses, suggesting that helper peptide would be a potent cancer vaccine without combination with killer and/or helper peptides [45, 46]. Additionally, easy formulation of a single helper peptide rather than multiple peptides vaccine should be an additional advantage of OCV-501 from drug development point of view. Also, OCV-501, single WT1-helper peptide, had helper peptide activities including wide-range HLA-class II restrictions and cytolytic CD4^+^ T cell-induction which might be stronger than that of the other TAA-helper peptides.

We conducted an open label, multi-center, phase 1 trial of OCV-501 to evaluate the safety and tolerability in older AML patients in CR. Nine patients were enrolled and all completed the study. All doses of OCV-501 administered subcutaneously 4 times every 4 weeks were well-tolerated and safe, and the MTD was considered to be ≥3 mg. Injection site reactions were observed in all patients at all dosage levels. These might have been due to the Montanide used as an adjuvant, the frequent side effects of which are well known to include inflammatory reactions, granulomas and ulcers at the injection site [47]. Clinical efficacies and immunological responses were unconfirmed in this clinical study. However, no patients relapsed during the study. All the patients provided informed consent to continue further vaccination with OCV-501 in an extension trial to evaluate the safety and efficacy of continuous administration of OCV-501. Immune response in DTH with induration of >5 mm diameter was observed in 2 patients, suggesting that these might be clues to clinical efficacy due to OCV-501 vaccination. Anti-OCV-501 IgG formation was found in rats and dogs in pre-clinical studies. Although anti-OCV-501 Ab was not detected during the study, this appeared in some patients in the follow-up period after the study (data not shown). These data might also support immune responses to OCV-501 suggesting the rationale to use the dose range of this study in future studies.

In conclusion, OCV-501, a WT1 helper peptide, induced OCV-501-specific Th1 responses dose-dependently and stimulated helper activity of the specific Th1 cells in PBMC from healthy donors in an HLA class II-restricted manner. OCV-501-specific Th1 clones showed significant OCV-501-specific cytolytic activity against B-LCL cells. In this phase 1 clinical trial, OCV-501, administered subcutaneously once a week for 4 weeks to older patients with AML, was well-tolerated and safe with a considerable MTD of ≥3 mg. Further clinical studies of OCV-501 in patients with AML should be considered to confirm its safety, and efficacy.

## Electronic supplementary material

Below is the link to the electronic supplementary material.

Supplementary material 1 (PDF 256 KB)

## Abbreviations

AML: Acute myeloid leukemia
B-LCL: B-lymphoblastoid cell line
CR: Complete remission
DLT: Dose-limiting toxicity
DTH: Delayed-type hypersensitivity
MTD: Maximum tolerated dose
TEAEs: Treatment-emergent adverse events
Th1: Type 1 T-helper
WT1: Wilms’ tumor 1
