# Supplementary material for: A new peptide vaccine OCV-501: in vitro pharmacology and phase 1 study in patients with acute myeloid leukemia
Source: Cancer Immunol Immunother. 2017 Mar 20;66(7):851–63. doi: 10.1007/s00262-017-1981-3 (PMC5489634; doi:10.1007/s00262-017-1981-3)
Supplement: Supplementary file 1 — Supplementary material 1 (PDF 256 KB) [file 262_2017_1981_MOESM1_ESM.pdf]

Supplementary

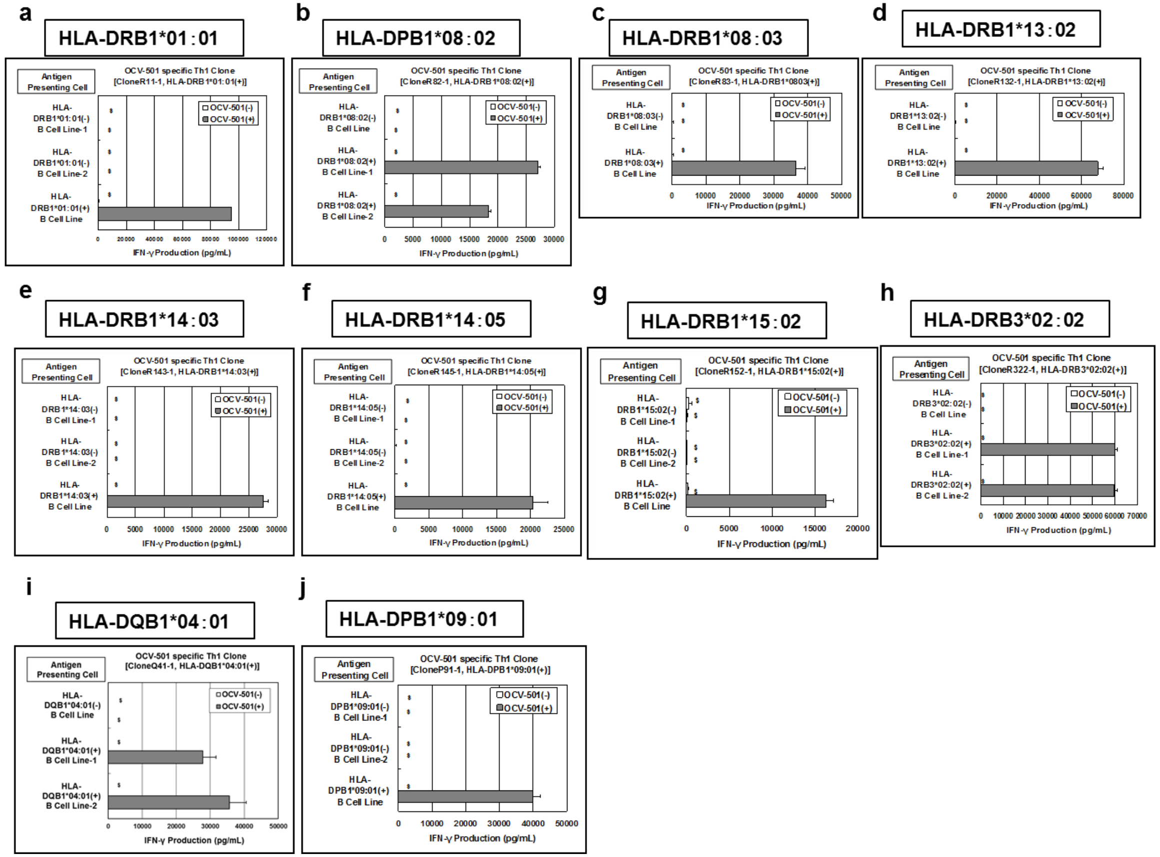

Supplementary Fig. 1 Efficacy pharmacology studies of OCV-501 using various T-cell clones

B-LCL: B-lymphoblastoid cell line, Th1: type 1 T-helper cell.

HLA-restricted T-cell activation by OCV-501 using various human T-cell clones. As an index of T-cell activating effect, the produced IFN-γ from OCV-501-specific Th1 clone was estimated in the culture of each HLA class II(+) or HLA class II(-) B-LCLs pulsed with or without OCV-501 [Clone R11-1: HLA-DRB1*01:01(+) (**a**), Clone R82-1: HLA-DRB1*08:02(+) (**b**), Clone R83-1: HLA-DRB1*08:03(+) (**c**), Clone R132-1: HLA-DRB1*13:02(+) (**d**), Clone R143-1: HLA-DRB1*14:03(+) (**e**), Clone R145-1: HLA-DRB1*14:05(+) (**f**), Clone R152-1: HLA-DRB1*15:02(+) (**g**), Clone R322-1: HLA-DRB3*02:02(+) (**h**), Clone Q41-1:HLA-DQB1*04:01 (**i**), and Clone P91-1: HLA-DPB1*09:01(+) (**j**)] [data are expressed as mean ± SD, triplicates, $: error bar was calculated by extrapolated data]

Supplementary Table 1 Bone marrow examination

Dose	Subject ID	Time Point	Nucleated Cell Count (10^4/uL)	Myeloblast (%)	Neutrophil (%)	Basophil (%)	Eosinophil (%)	Monocyte (%)	Lymphocyte (%)	Atypical Lymphocyte (%)
0.3 mg	001-0001	Screening	4.580	1.0	29.2	0.2	4.4	5.2	18.2	0.0
		End of Treatment	2.600	1.0	29.2	0.8	4.6	7.4	22.4	0.0
	002-0001	Screening	33.875	0.7	41.5	0.0	2.0	3.0	23.7	0.0
		End of Treatment	60.150	1.0	41.2	0.0	0.2	0.3	12.0	0.0
	002-0002	Screening	4.800	0.8	23.6	0.0	2.0	2.0	34.8	0.0
		End of Treatment	7.325	1.7	31.0	0.0	1.7	6.7	24.7	0.0
1.0 mg	001-0002	Screening	2.310	1.2	35.0	0.6	5.0	9.2	21.8	0.0
		End of Treatment	3.060	0.8	24.6	0.4	8.4	6.0	17.2	0.0
	002-0003	Screening	4.800	1.2	40.8	0.0	2.4	6.2	18.4	0.0
		End of Treatment	2.650	2.4	27.2	0.0	2.4	5.6	14.4	0.0
	002-0004	Screening	2.034	1.2	34.2	0.0	0.8	5.0	12.6	0.0
		End of Treatment	18.500	1.6	32.8	0.0	5.6	4.8	12.0	0.0
3.0 mg	001-0003	Screening	7.300	1.0	34.6	0.4	1.0	2.2	11.0	0.0
		End of Treatment	0.490	0.0	36.5	0.5	1.0	7.0	37.0	0.0
		Unscheduled	9.330	0.8	31.6	0.0	0.4	1.6	11.2	0.0
	001-0004	Screening	17.140	0.4	32.2	0.0	0.2	1.4	9.8	0.0
		End of Treatment	4.310	3.0	28.4	0.2	0.8	7.2	14.0	0.0
	002-0005	Screening	1.650	2.8	44.0	0.0	0.8	2.8	8.0	0.0
		End of Treatment	4.8750	1.2	43.2	0.0	5.6	4.0	7.6	0.0

Supplementary Table 2 Specific Delayed-type Hypersensitivity Reaction after OCV-501 Administration

Dose	Subject ID	Time Point	Redness (mm)	Induration (mm)
0.3 mg	001-0001	Screening	0	0
		End of Treatment	2	0
	002-0001	Screening	0	0
		End of Treatment	1	0
	002-0002	Screening	0	0
		End of Treatment	7	7
1.0 mg	001-0002	Screening	1	0
		End of Treatment	4	0
	002-0003	Screening	0	0
		End of Treatment	0	0
	002-0004	Screening	0	0
		End of Treatment	0	0
3.0 mg	001-0003	Screening	0	0
		End of Treatment	1	0
	001-0004	Screening	0	0
		End of Treatment	0	0
	002-0005	Screening	0	0
		End of Treatment	6	8

Supplementary Table 3 Immunoglobulin G (IgG) level

Dose	Subject ID	Time Point	Time of Blood Collection	IgG Level (mg/dL)
0.3 mg	001-0001	Screening	9:59	1387
		End of Treatment	10:47	1601
		Post Treatment	9:20	1725
	002-0001	Screening	8:10	1097
		End of Treatment	10:49	1041
		Post Treatment	9:40	1106
	002-0002	Screening	8:00	1499
		End of Treatment	10:52	1582
		Post Treatment	9:27	1417
1.0 mg	001-0002	Screening	8:17	1452
		End of Treatment	11:11	1437
		Post Treatment	11:10	1394
	002-0003	Screening	8:13	1410
		End of Treatment	11:01	1336
		Post Treatment	11:10	1330
	002-0004	Screening	12:10	1327
		End of Treatment	11:27	1266
		Post Treatment	13:39	1440
3.0 mg	001-0003	Screening	12:03	1140
		End of Treatment	13:07	1285
		Post Treatment	12:08	1224
	001-0004	Screening	6:58	1403
		End of Treatment	10:55	1271
		Post Treatment	10:56	1298
	002-0005	Screening	9:14	1499
		End of Treatment	11:45	1522
		Post Treatment	11:38	1491
